# Stain SAN: simultaneous augmentation and normalization for histopathology images

**DOI:** 10.1117/1.JMI.11.4.044006

**Published:** 2024-08-23

**Authors:** Taebin Kim, Yao Li, Benjamin C. Calhoun, Aatish Thennavan, Lisa A. Carey, W. Fraser Symmans, Melissa A. Troester, Charles M. Perou, J. S. Marron

**Affiliations:** a University of North Carolina at Chapel Hill, Department of Statistics and Operations Research, Chapel Hill, North Carolina, United States; b University of North Carolina at Chapel Hill, Department of Pathology and Laboratory Medicine, Chapel Hill, North Carolina, United States; c University of North Carolina at Chapel Hill, UNC Lineberger Comprehensive Cancer Center, Chapel Hill, North Carolina, United States; d The University of Texas MD Anderson Cancer Center, Department of Systems Biology, Houston, Texas, United States; e University of North Carolina at Chapel Hill, Department of Medicine, Chapel Hill, North Carolina, United States; f The University of Texas MD Anderson Cancer Center, Department of Pathology, Houston, Texas, United States; g The University of Texas MD Anderson Cancer Center, Department of Translational Molecular Pathology, Houston, Texas, United States; h University of North Carolina at Chapel Hill, Department of Epidemiology, Chapel Hill, North Carolina, United States; i University of North Carolina at Chapel Hill, UNC Center for Environmental Health and Susceptibility, Chapel Hill, North Carolina, United States; j University of North Carolina at Chapel Hill, Department of Genetics, Chapel Hill, North Carolina, United States; k University of North Carolina at Chapel Hill, Department of Biostatistics, Chapel Hill, North Carolina, United States

**Keywords:** batch adjustment, color transformation, domain adaptation, histopathology, tissue staining

## Abstract

**Purpose:**

We address the need for effective stain domain adaptation methods in histopathology to enhance the performance of downstream computational tasks, particularly classification. Existing methods exhibit varying strengths and weaknesses, prompting the exploration of a different approach. The focus is on improving stain color consistency, expanding the stain domain scope, and minimizing the domain gap between image batches.

**Approach:**

We introduce a new domain adaptation method, Stain simultaneous augmentation and normalization (SAN), designed to adjust the distribution of stain colors to align with a target distribution. Stain SAN combines the merits of established methods, such as stain normalization, stain augmentation, and stain mix-up, while mitigating their inherent limitations. Stain SAN adapts stain domains by resampling stain color matrices from a well-structured target distribution.

**Results:**

Experimental evaluations of cross-dataset clinical estrogen receptor status classification demonstrate the efficacy of Stain SAN and its superior performance compared with existing stain adaptation methods. In one case, the area under the curve (AUC) increased by 11.4%. Overall, our results clearly show the improvements made over the history of the development of these methods culminating with substantial enhancement provided by Stain SAN. Furthermore, we show that Stain SAN achieves results comparable with the state-of-the-art generative adversarial network-based approach without requiring separate training for stain adaptation or access to the target domain during training. Stain SAN’s performance is on par with HistAuGAN, proving its effectiveness and computational efficiency.

**Conclusions:**

Stain SAN emerges as a promising solution, addressing the potential shortcomings of contemporary stain adaptation methods. Its effectiveness is underscored by notable improvements in the context of clinical estrogen receptor status classification, where it achieves the best AUC performance. The findings endorse Stain SAN as a robust approach for stain domain adaptation in histopathology images, with implications for advancing computational tasks in the field.

## 1 Introduction

In histopathology, stained microscopic images such as whole slide images (WSIs) and tissue microarrays (TMAs) are scanned to be examined by pathologists or to be fed to computer-aided diagnosis models. Hematoxylin and eosin (H&E) staining is one of the most common staining methods in the field. Hematoxylin stains acidic structures, including DNA, imparting a blue-purple color to the nucleus in standard light microscopy. Basic structures, including cytoplasmic proteins and collagen in the stroma, are stained orange–red–pink with eosin. However, differences due to effects such as staining protocols,^1^ solution preparation procedures,^2^ different scanners,^3^ and aging could cause unwanted color variation across slide images, as shown in the top row of Fig. 4, which hampers the performance of population-level downstream tasks, including classification. Therefore, reducing potential undesirable differences among stain colors and obtaining robust color representations are imperative steps in the preprocessing of histology images. We call this process stain adaptation.

Past stain adaptation methods include those based on matrix decomposition [e.g., singular value decomposition (SVD)^4^ and non-negative matrix factorization^5^^,^^6^] and those based on deep learning.^7^^–^^10^ The deep learning approaches require training separate neural networks, which are often computationally expensive. Despite the appeal of deep learning, Tellez et al.^7^ showed that a matrix decomposition-based stain adaptation can perform at least as well as current existing deep learning approaches in multiple classification tasks. Thus, we focus here on matrix decomposition methods to save computational resources without significant loss of performance.

Throughout this paper, we consider an RGB color image with d pixels as a data object, and each image is transformed to the optical density (OD) space (the more useful color scale of the negative log of RGB) following the Beer–Lambert law^11^ for color representation. Denote the OD-transformed image as $V\in R^{3\times d}$. As described by Vahadane et al.,^6^ the common first step of stain adaptation is a useful decomposition of the stained image into a stain color matrix W with m (2 or 3) color vectors and a stain intensity matrix H as

$$V=WH,\;W\in R^{3\times m},\;H\in R^{m\times d}.$$ (1)

Most methods studied here focus on the case of m=2 because H&E staining employs two colors in the staining process. In particular, one column vector in W represents hematoxylin and the other represents eosin.

**Fig. 1:**
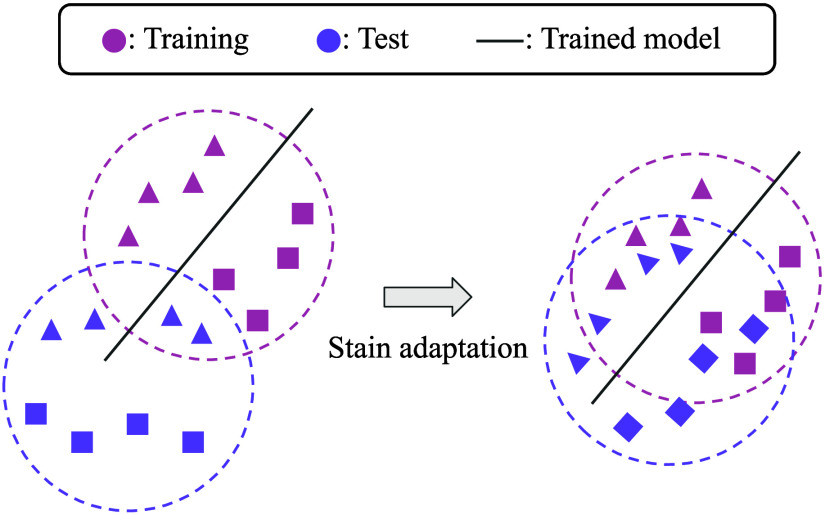
Stain domains (probability distributions) are represented by dashed circles. Different shapes represent classification labels. Colors represent training versus test images. Notice the gap between training and test domains. The classification model trained on the left may not properly classify the test images due to the domain gap between the training and test domains. Various stain adaptation methods aim to reduce the domain gap as shown on the right and thus provide improved classification validation.

Then, we define the notion of stain domain as a probability distribution representation of the set of potential stain color and intensity matrices. When separate datasets are given for training and testing a machine learning model, differences between stain domains can cause loss of generalizability of the model, as shown on the right side of Fig. 1.^7^^,^^12^ On the left side of Fig. 1, notice that the model that learned on the training group without stain adaptation gives inaccurate classification of the test group and hence poor validation. A major contribution of this paper is developing a new stain adaptation method. It helps the model on the right side perform better by well adapting the stain domains where the major improvement comes from reducing stain domain gaps.

**Fig. 2:**
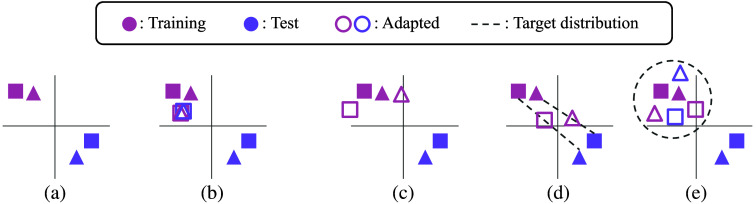
Illustration of the stain color matrix W, which contrasts different stain adaptation methods. Different shapes represent classification labels. Solid shapes represent the original matrices in $R^{3\times m}$ in each panel, and the corresponding hollow symbols represent the adapted matrices. Dashes represent target stain domains (probability distributions). (a) Original. (b) Stain normalization. (c) Stain augmentation. (d) Stain mix-up. (e) Stain SAN.

Early approaches to stain adaptation are called stain normalization.^4^^–^^6^^,^^12^ Such methods alleviate color variation by normalizing the stain colors of all stained images to a common target color set. As shown in Fig. 2, stain color matrices W of both training and test images are replaced by a common reference matrix. The overlapping hollow symbols in Fig. 2(b) represent the same reference stain color matrix. A common practice is to rescale the stain intensity matrices H so that each row has the same say 99th percentile across images. Stain normalization reduces any domain gaps by linearly transforming all domains to a narrower target domain. Macenko et al.^4^ utilized singular value decomposition to calculate image-specific stain matrices on OD space. Khan et al.^5^ took a non-linear mapping approach to stain normalization. Vahadane et al.^6^ employed sparse non-negative matrix factorization (SNMF). Nadeem et al.^12^ used the Wasserstein barycenter. Although stain normalization improves the visual impression and consistency of images, it generally decreases the diversity of the stain domain as the target domain is limited to a fixed stain color matrix. Such domain shrinkage can give less effective performance in some downstream tasks.

An interesting alternative approach is stain augmentation.^7^^,^^13^ The goal of stain augmentation is to bring down domain gaps between the target and source domains by transforming the target domain into a larger distribution. This extends the generalizability of a model. As in Fig. 2(c), the stain color matrices W of training images are perturbed in the $R^{3\times m}$ space. Tellez et al.^13^ decomposed images into three fixed color channels using the stain deconvolution described in Ref. 14. They then perturbed stain intensities of each channel by adding and multiplying by uniform random variables, with an independent realization for each image. Consider the case of m=3 in Eq. (1). Then, for i=1,2,3 and small positive values of $\varepsilon_{1}$ and $\varepsilon_{2}$, each color channel H(i) was randomly perturbed as

$$\alpha_{i}\cdot H(i)+\beta_{i},$$ (2)

$$\alpha_{i}∼\text{Uniform}(1-\varepsilon_{1},1+\varepsilon_{1}),\;\beta_{i}∼\text{Uniform}(1-\varepsilon_{2},1+\varepsilon_{2}),$$

where each image object was augmented with a separate realization of $\alpha_{i}$ and $\beta_{i}$. Note that this provides an equivalent output matrix as perturbing each column of W, i.e., transforming the i’th column W(i) to

$$\alpha_{i}\cdot W(i)+\beta_{i}.$$ (3)

Alternate approaches include Ref. 15, which perturbed principal components of stained images, and Ref. 16, which employed Bayesian modeling for stain color deconvolution. Chang et al.^17^ showed that perturbation of stain matrices obtained by SNMF stain extraction significantly improves the performance of tumor classification. Tellez et al.^7^ demonstrated that stain augmentation outperforms stain normalization for multiple classification tasks. However, stain augmentation does not always effectively shrink the domain gap between batches of images. Perturbation of stain matrices may not guarantee the domain gap reduction as there is no established target distribution.

Stain mix-up^17^ is a stain domain adaptation method based on stain extraction that achieves state-of-the-art results in improving different downstream tasks. It is based on mix-up,^18^ which is a popular data augmentation and domain adaptation method in computer vision. Stain mix-up aims to mix the target and source domains using randomly interpolated stain color matrices. In particular, given an OD-transformed image $V_{j}$ from the source dataset, another OD image object $V^{k}$ is randomly chosen from the target dataset. As in Eq. (1), let $W_{j}$ and $H_{j}$ be the stain color matrix and stain intensity matrix of $V_{j}$ and let $W^{k}$ be the stain color matrix of $V^{k}$. Then, the corresponding target image is reconstructed with a randomly interpolated stain color matrix $W_{j}^{k}$ obtained as

$$W_{j}^{k}=(1-u)\cdot W_{j}+u\cdot W^{k},$$ (4)

u∼Uniform(0,1),

and a randomly perturbed stain intensity matrix

$$\alpha \cdot H_{j},$$ (5)

α∼Uniform(1−ε,1+ε),

for a small positive value ε. Note that Eq. (5) uses a single α across different color channels, whereas Eq. (2) samples one set of $\alpha_{i}$ and $\beta_{i}$ for each color channel H(i). A graphical representation of this step is presented in Fig. 2(d). Note that the dashed lines show the target stain domains for random interpolation from which the mixed matrices are sampled. Despite its success in improving the performance of multiple downstream tasks, stain mix-up can be an overly optimistic method in the sense of requiring access to the test set during training time. This can reduce the validity of test accuracy. Furthermore, stain mix-up needs to be reimplemented every time a new dataset is encountered, which can increase the computational burden in both experiments and analyses.

**Fig. 3:**
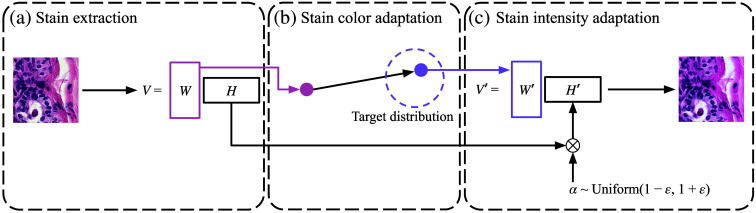
Diagram showing the three steps of Stain SAN. (a) Stained image is first transformed to OD space, and the stain matrices are extracted. (b) Stain color matrix W is resampled from the target distribution. (c) Stain intensity matrix H is perturbed by multiplying a uniform random variable, and the adapted image is reconstructed.

We propose a novel stain domain adaptation method, Stain SAN, which merges stain color distributions of different datasets with the guarantee of domain gap reduction. Furthermore, Stain SAN is not overly optimistic. The model reduces the domain gap by transforming different domains into a reasonable target domain. As shown in Fig. 3, Stain SAN is comprised of three steps: (a) stain extraction, (b) stain color adaptation, and (c) stain intensity adaptation. First, each given stained image I is transformed to an OD object V, which is decomposed into the stain color matrix W times the stain intensity matrix H as in Eq. (1). See Fig. 2 for comparison to other stain adaptation methods. In stain color adaptation, the stain color matrix W is resampled from the target distribution that is determined by the training images. Finally, in stain intensity adaptation, the stain intensity matrix H is perturbed by multiplying a random uniform variable as in Eq. (5), and the stain-adapted image is reconstructed. More details are discussed in Sec. 2. To the best of our knowledge, Stain SAN is the first domain adaptation method for histopathology images that generalizes the distribution of stain colors to the target distribution without relying on deep learning.

Section 3 shows the capability of Stain SAN by comparing its performance with other stain adaptation methods. These results emphasize the strength of Stain SAN over the other methods. Moreover, Stain SAN delivers outcomes comparable with the deep learning-based method without needing separate training for stain adaptation nor access to the target domain during training. Stain SAN’s performance is equivalent to one of the state-of-the-art methods, demonstrating its efficacy and efficiency.

## 2 Methods

In this section, we describe the details of our Stain SAN. It is composed of three steps as noted in Sec. 1. The first step is stain extraction using SVD as detailed in Fig. 3(a). Note that other decomposition methods including SNMF can also be used for extracting stain matrices. The second step is stain color adaptation by resampling the stain color matrix W in Eq. (1) from a proper target distribution, as represented in Fig. 3(b). The last step is stain intensity adaptation, which involves random perturbation of the stain intensity matrix H, as depicted in Fig. 3(c). Note that stain normalization, stain augmentation, and stain mix-up are usefully understood as special cases of Stain SAN. Our main contribution is that Stain SAN combines the best aspects of each. See Sec. 2.3 for more detail.

### 2.1 Stain Extraction

Given an RGB color image $I\in R^{3\times d}$, each color pixel is first converted to an OD pixel by applying the Beer–Lambert transformation^19^ as follows. Let $I_{0}$ be the incident luminous intensity. Then, the OD-transformed image object is

$$V=-\log\;\frac{I}{I_{0}}.$$ (6)

Note that a common practice is to collect 8-bit images and set $I_{0}=255$.

We decompose V into the stain color matrix W and the stain intensity matrix H following the SVD-based stain extraction method in Ref. 4. After transforming the images following Eq. (6), background OD pixels are masked based on the distance from the origin. During the masking process, any pixel whose distance exceeds 0.3 is hidden when determining stain domains, and these hidden pixels are recovered when generating the adapted images. The threshold of 0.3 was selected following manual observation of the polar-coordinate plot representing stain color intensity on the two-dimensional plane defined by the stain color vectors.

### 2.2 Energy-Based Stain Adaptation

In Stain SAN, the stain color matrix W is resampled from the target stain color distribution, and this combines the strengths of previous stain adaptation methods. First, Stain SAN improves the consistency of the images by adapting both target and source batches. Second, Stain SAN increases the diversity of stain domains by perturbing the stain color matrix and the stain intensity matrix. Third, Stain SAN reduces the gap between different stain domains by replacing the distribution of the stain colors with a target distribution.

Stain SAN simultaneously augments and normalizes histopathology images by adapting stain color distributions to a target distribution. To facilitate covariance calculation, it is convenient to let $\overset{¯}{W}\in R^{3m}$ denote the reshaped column vector of the corresponding stain color matrix W. We model the target stain distribution as a Gaussian distribution based on the original batch of training images. The mean of the distribution is taken to be the element-wise median of the stain color matrices in the batch, ${\overset{¯}{W}}_{0}$. Then, the variance of the target distribution is computed using the covariance matrix of the training stain color matrices

$$\mathrm{Cov}(\overset{¯}{W})=E\{[\overset{¯}{W}-E(\overset{¯}{W})]{\left[\overset{¯}{W}-E(\overset{¯}{W})\right]}^{T}\}.$$ (7)

The trace of $\mathrm{Cov}(\overset{¯}{W})$ represents the total energy of the distribution. The target stain distribution is taken to be the spherical Gaussian distribution that preserves the energy of the original distribution in the training set. Recalling that the dimension of the stain color matrix W is 3×m, the variance term $\sigma^{2}$ is chosen to satisfy

$$3\cdot m\cdot \sigma^{2}=\text{Trace}(\mathrm{Cov}(\overset{¯}{W})).$$ (8)

Then, $\bar{W}$ for the training images are resampled from the Gaussian distribution

$$N_{3}({\bar{W}}_{0},\sigma^{2}I_{3m}),$$ (9)

where $I_{n}$ represents the n×n identity matrix, and $\bar{W}$ for the test images is normalized to ${\bar{W}}_{0}$. We use a Gaussian distribution to have a better structured and simpler target domain, and we resample the test stain color matrices with zero variance so that they are not random and are better centered at $W_{0}$.

After resampling the stain color matrix W, the stain intensity matrix H is perturbed following Eq. (5). Then, the adapted image is reconstructed by transforming the image object back to the RGB space. Denoting $W^{\prime }$ and $H^{\prime }$ as these adapted stain color and stain intensity matrices, respectively, the adapted image $I^{\prime }$ is obtained by inverting the Beer–Lambert transformation^19^ as

$$I^{\prime }=I_{0}\;\exp(-W^{\prime }H^{\prime }),$$ (10)

for use in downstream tasks.

### 2.3 Stain SAN Benefits

As discussed in Sec. 2.2, the benefit of Stain SAN is that it combines the strengths of the previous stain adaptation methods while overcoming the weaknesses of the methods. Table 1 summarizes the relative strengths and weaknesses of different stain adaptation methods. Stain normalization aligns stain color across groups of images and guarantees domain gap reduction by replacing the stain color matrix W with the target matrix, but it has less capacity for domain generalization as the target distribution is fixed to the single $W_{0}\in R^{3\times m}$. Stain augmentation provides a broader stain domain by perturbing W, but it has the potential to produce less relevant perturbations. There is also no guaranteed reduction in the domain gap. Stain mix-up partially aligns stain colors, generalizes the stain domain, and provides a guaranteed reduction in domain gap by replacing W with a randomly interpolated stain color matrix, as in Eq. (4). However, it makes use of the other batch of images at the time of stain adaptation, which is infeasible in some realistic scenarios. For instance, when the goal is to train a generalized model that can be later applied to new external test datasets, stain mix-up fails to provide a reproducible training pipeline as it requires recalculation of the stain matrices whenever an external group of images is introduced, thus invalidating the conventional independent data testing process.

**Table 1:** Properties of stain adaptation methods.

Method	Color alignment	Domain generalization	Domain gap reduction	No use of external data
Stain normalization	+	−	+	+
Stain augmentation	−	+	−	+
Stain mix-up	∼	+	+	−
Stain SAN	+	+	+	+

The columns represent important properties. The symbols +, ∼, and −, represent positive, partial, and negative properties, respectively. This shows that only Stain SAN has all positive properties.

Stain SAN performs stain color alignment by resampling the stain color matrix W from a target domain that is more condensed than the union of the training and test domains. The stain domain is also generalized in the sense that the target distribution has positive variance and the total energy of the distribution of the stain color matrix W is the same as the original training domain. There is also a guarantee in domain gap reduction because W is adjusted for both training and test datasets. Moreover, it does not make use of external test images at training time because the target distribution is determined using the training group.

The flexibility of the Stain SAN framework means that the previous methods are special cases. In particular, depending on the target domain, each stain normalization, stain augmentation, and stain mix-up can be considered a form of Stain SAN. Table 2 shows the corresponding target domains of the stain color matrix W for the training and test batches. Let $\delta_{x}$ denote the Dirac delta measure on a point x, i.e.,

$$\delta_{x}(A)=\{\begin{array}{cc} 1 & \text{if}\;x\in A\\ 0 & \text{otherwise}, \end{array}$$ (11)

and recall that $W_{0}$ is the mean of the Gaussian target distribution in Sec. 2.2. Then, stain normalization is a form of Stain SAN where the training and test target domains for the stain color matrix W are both $\delta_{W_{0}}$. Stain augmentation can be considered a special case of Stain SAN with the uniform perturbation for each image in Eq. (3) as the training target domain. The test target domain should be $\delta_{W}$ as the test stain matrices are not changed. For stain mix-up, Eq. (4) provides the target domain for both the training and test groups.

**Table 2:** Previous stain adaptation methods as special cases of Stain SAN.

Method	Training target domain	Test target domain
Stain normalization	$\delta_{W_{0}}$	$\delta_{W_{0}}$
Stain augmentation	Eq. (3)	$\delta_{W}$
Stain mix-up	Eq. (4)	Eq. (4)

The target distribution of the stain color matrix W for each method is listed. $\delta_{x}$ represents the Dirac delta measure on x.

## 3 Experimental Results

In this section, we discuss the comparison of different stain adaptation methods on some histopathology image datasets. We evaluated the performance of the methods on the classification of the important diagnostic indicator of estrogen receptor (ER) status.

### 3.1 Datasets

The two datasets used in the experiment were obtained from two different breast cancer patient sets: the Cancer and Leukemia Group B 9741 (CALGB 9741)^20^ and the Carolina Breast Cancer Study (CBCS).^21^ The histopathology images provided in the studies were TMA core images at 20× magnification. These are circular disks of less than 1 mm in diameter from core tissue samples. Table 3 shows the detailed characteristics of the datasets.

**Table 3:** Dataset characteristics.

Dataset	n subjects	n cores	Core size (mm)	ER 10%
CALGB 9741	990	2007	0.6	+533/−457
CBCS	1436	3563	1.0	+1018/−418

**Fig. 4:**
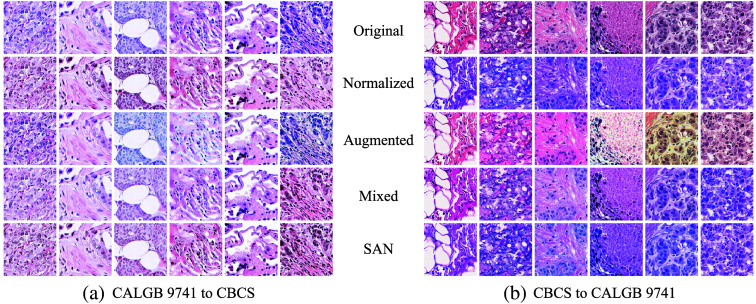
Example of adapted TMA patches. From top to bottom, each row shows original, normalized-, augmented-, mixed-, and SAN-adapted images in order. In panel (a), images from CALGB 9741 are adapted with CBCS as the other group. Panel (b) shows the reversed case. Stain SAN gives the best result.

The stained images were collected from different labs in different time frames; thus, the stain domain gap between the image groups should be taken into account when training and testing models using the images. The first row in Fig. 4 shows systematic color variation across the groups. In particular, there tend to be stronger purple on the left (CALGB 9741) and a more reddish hue on the right (CBCS). The other rows illustrate the effect of different stain adaption methods on these stained images. See Sec. 3.3 for a detailed description.

As noted above, an important goal is the classification of clinical ER status using the H&E images. Clinical ER status is obtained from an expert pathologist’s visual examination of ER immunohistochemistry (IHC) stained images.^22^ Although IHC staining is precise, there is a substantial added cost over the standard H&E staining, which motivates machine learning-based prediction of clinical ER status from H&E images.^23^ IHC staining methods stain target antibodies brown and other cellular structures blue. A tissue image is labeled ER positive if the proportion of brown stained cells is higher than the cutoff and negative if it is lower than the cutoff. The 10% threshold was used for clinical ER status in CALGB 9741 and CBCS as this was the clinical definition when these samples were collected.

### 3.2 Implementation Details

We evaluated the performance of different stain adaptation methods described in Sec. 1: stain normalization,^4^ stain augmentation,^13^ stain mix-up,^17^ and our proposed method Stain SAN. These methods were compared based on a downstream classification task.

We followed the multiple instance learning classification model framework described in Ref. 24. First, each TMA core image was split into 400×400 (in pixel dimensions) non-overlapping patches. Then, they were input to the pretrained VGG16^25^ for extracting 512-dimensional patch features. Next, we trained a patch-level support vector machine (SVM) classifier^26^ using the patch features and aggregated 16 equally spaced quantiles of the probability outcomes of the trained SVM. Finally, we trained another patient-level SVM classifier using the aggregated quantiles.

For model validation, we obtained the mean area under the curve (AUC) along with its corresponding standard error by training the classification model on a cross-dataset validation setting. In this setting, both the training and test datasets were split into five equal-sized partitions, where four of the training partitions and one test partition were used for training and validation. Similar to the traditional cross-validation technique,^27^ this step was repeated five times, and the left-out groups in the training data did not overlap with the included groups in the test data across the iterations. This design ensured independent estimates of the AUC, which gave valid standard errors of the mean over iterations.

To obtain comparable results over stain adaptation methods, the parameters $\varepsilon_{1}$ and $\varepsilon_{2}$ in Eq. (2) and ε in Eq. (5) were all set to 0.2. Note that these parameters correspond to the optimal parameters chosen in Refs. 7 and 17. The optimal penalty parameters C of the patch-level and patient-level SVM classifiers were determined using 20-fold cross-validation over the grid $\left\{2^{-15},2^{-14},\ldots ,2^{10}\right\}$.^28^ The SVM classifiers were trained using samples that were inversely weighted according to the class proportions.^29^

### 3.3 Results

Figure 4 presents example images of adapted TMA core patches. Original images without manipulation are included in the first row. Normalized-, augmented-, mixed-, and SAN-adapted images are shown in the second, third, fourth, and fifth rows, respectively. Figure 4(a) represents adapted CALGB 9741 cores, and Fig. 4(b) shows adapted CBCS cores. The properties of different adaptation methods discussed in Table 1 can be observed in the figure. For stain normalization, the test images adapted to the color basis of the training images are included in the second row. Note that the stain colors of the images in each block are better adapted to the colors of the images in the other block, but there is less variability in color within each group of images. The augmented training images are shown in the third row. As each stain color matrix is perturbed with a random noise, we can observe a wider range of stain colors. Furthermore, the images in the first row of one block and the third row of the other block do not share a similar hue. In the fourth row, the mixed training images are visualized in each block. The images show how random interpolation in stain mix-up generalizes stain domains with guaranteed domain gap reduction. The adapted stain colors are well located on the spectrum between the two sets of colors at the cost of using the test images at the training time. The last row represents the corresponding Stain SAN adapted images, which profit from improved color alignment, generalized stain domains, and domain gap reduction.

**Fig. 5:**
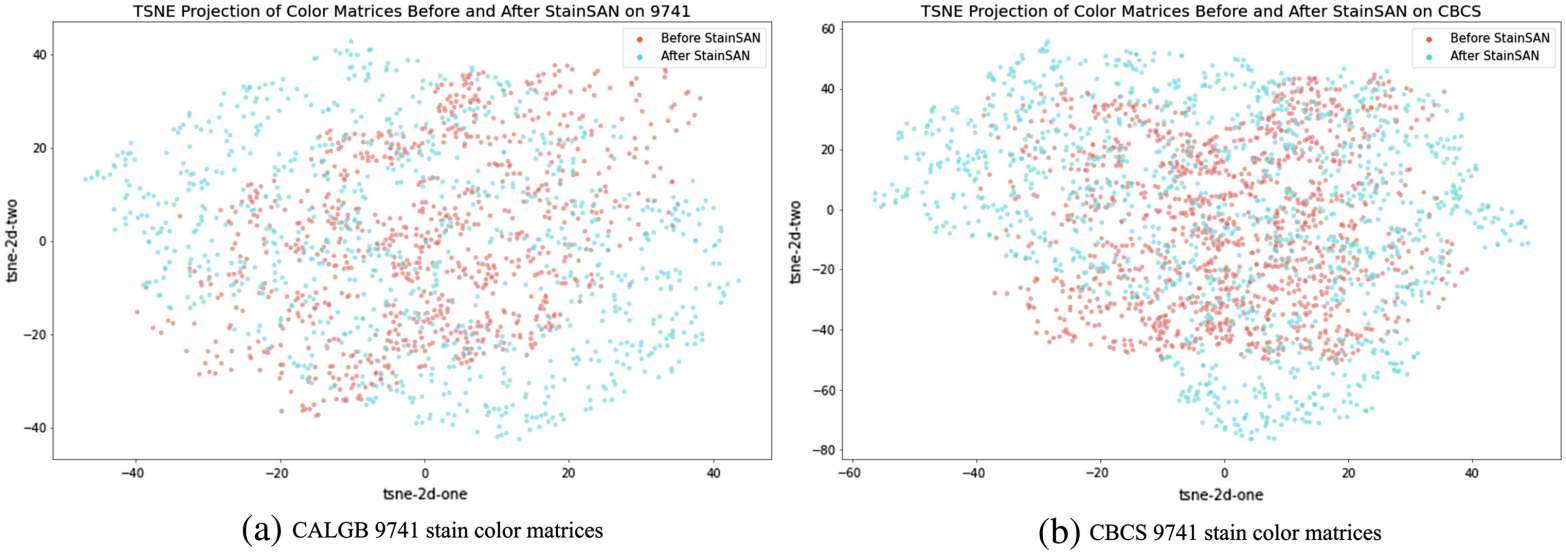
TSNE projection of stain color matrices before and after applying Stain SAN.

Figure 5 exhibits the stain color generalization by visualizing t-distributed stochastic neighbor embedding (t-SNE)^30^ projection of stain color matrices before and after applying Stain SAN. It is shown that Stain SAN generalizes the stain color domain for both CALGB 9741 and CBCS by resampling the stain color matrices from the reconstructed Gaussian distribution described in Eq. (9).

**Fig. 6:**
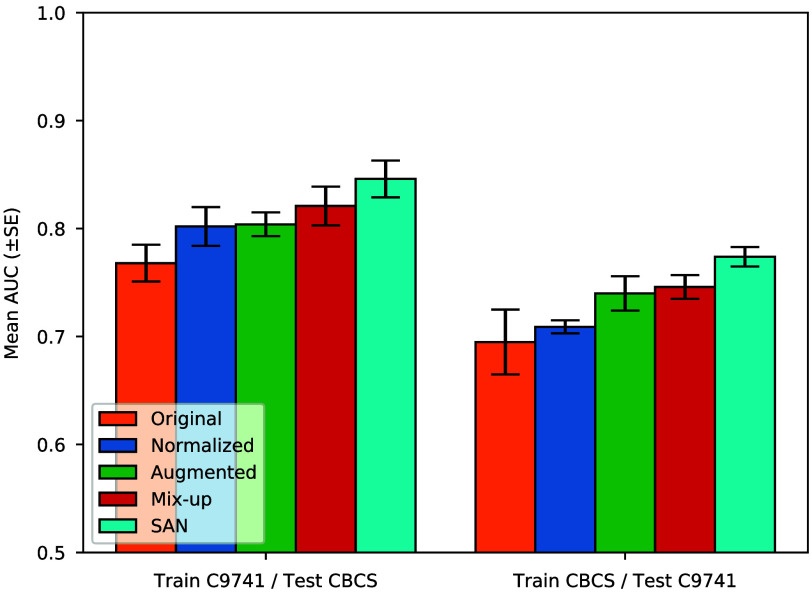
Mean AUC (±SE) on two classification tasks in five distinct training and testing scenarios: without stain adaptation (original), with stain normalization, with stain augmentation, with stain mix-up, and with Stain SAN. The left side presents the models trained on CALGB 9741 and tested on CBCS. The right side exhibits the models trained on CBCS and tested on CALGB 9741. Historical improvements over time, with Stain SAN obtaining the best results.

The comparison results of cross-dataset ER 10% status classification using H&E TMA images are presented in Fig. 6. When there is no manipulation, the mean AUC (±SE) of the classification model is 0.768 (±0.017) when trained on CALGB 9741 and 0.695 (±0.030) when trained on CBCS. Stain normalization increases the former and latter AUCs by 0.034 and 0.014, respectively. When stain augmentation is applied, the trained models give higher mean AUCs, and the error bars do not overlap for the latter task. The models trained with stain mix-up further improve the mean AUCs. Stain SAN provides the mean AUCs of 0.846 (±0.017) and 0.774 (±0.009), which are the highest in both cases. The stain adaptation methods show a gradual boost in the performance of the machine learning classification models as they steadily improved and that our method gives the best overall results.

**Fig. 7:**
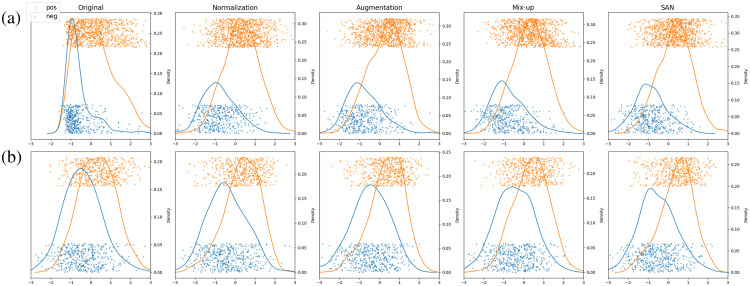
Cross-dataset [(a) trained on CALGB 9741/tested on CBCS, (b) trained on CBCS/tested on CALGB 9741] scores of the trained patient-level SVM. Columns represent different stain adaptation methods. Orange and blue indicate ER positive and ER negative, respectively. Dots represent patients and curves show their kernel density estimates. The gradual improvement in separation of the two classes from the left to the right.

The patient-level outcomes of the SVM classifiers trained on one dataset and tested on the other are shown as colored dots in Fig. 7. We call the standardized values of these outcomes cross-dataset scores with distributions visualized in Fig. 7. The first row shows the model trained on CALGB 9741 and tested on the independent CBCS dataset. The second shows training on CBCS and testing on CALGB 9741. The columns present the results of the models trained on the images adapted with different methods. The methods are listed in chronological order from left to right, demonstrating the improvements made by each. ER-positive and -negative patients are represented by orange and blue dots, respectively. The curves with corresponding colors are kernel density estimates (i.e., smooth histograms). It is shown that the more stain adaptation methods provide better separation of the two classes on the SVM classification axes. In particular, there is a gradual improvement over time in the separation of the distributions (see the increasing distance between the density peaks) as well as in AUC.

### 3.4 Target Stain Distribution

To enable the application of Stan SAN at the single image level and to extend the reproducibility of our method, we provide the target stain distribution obtained as in Sec. 2.2 using the CBCS H&E images. The total energy-preserving Gaussian distribution in the OD space is given by Eq. (9) with

$$W_{0}=[\begin{array}{cc} 0.544 & 0.141\\ 0.703 & 0.821\\ 0.455 & 0.552 \end{array}],\;\sigma =0.053.$$ (12)

Note that the training and test stain distributions in our experiment were $N({\overset{¯}{W}}_{0},\sigma^{2}I_{3m})$ and $N({\overset{¯}{W}}_{0},0I_{3m})$, respectively. This reference distribution is suitable for applying stain adaptation at both the individual image level and the batch level.

### 3.5 Impact on Expert Pathology

The outcomes of different stain adaptation methods presented in Fig. 4 have been assessed by a dedicated breast pathologist. Each row in Fig. 4 visualizes variation in color representation across different TMA cores. Each column of Fig. 4 shows different stain adaptation methods applied to each image. In most instances, variability in the color distribution did not dramatically affect the assessment of the tissue architecture or visualization of the relationship of the tumor cells to the stroma. Nuclear features, including the chromatin pattern, mitotic figures, and nucleoli, were relatively similar across the stain adaptation methods.

Stain normalization and Stain SAN (the second and fifth rows, respectively) were the most similar to each other and showed the most consistent color representation across different TMA cores. Stain augmentation (the third row) showed the largest diversity in color representation across different TMA cores in Fig. 4(b). This variability resulted in some images that closely resembled the original stain colors and some images with color distributions that diverged significantly from the original images and the other adapted images [e.g., the fifth column in Fig. 4(b)]. Overall, stain normalization and Stain SAN showed the most coherent contrast between the nucleus and cytoplasm and between the tumor and stroma. Stain augmentation resulted in a significant alteration in the color of cytoplasmic contents in some images [e.g., the third row of the third column in Fig. 4(a)].

### 3.6 Comparison with the GAN-Based Approach

In this section, we demonstrate that Stain SAN can achieve powerful results that are comparable with the state-of-the-art generative adversarial network (GAN)-based approach although it does not require a separate training for stain adaptation nor access to the target domain during training.

For benchmarking, we utilized the CAMELYON17 dataset, which provides WSIs from five different medical centers. Pixel-wise annotations are available for a total of 50 WSIs, with 10 from each center. For simplicity, our experiments focused on three centers denoted as centers 0, 1, and 2. Annotated slides (30 in total) from these centers were used in our experiments. Tumor and normal patches were extracted from the 30 annotated slides. The patches from each center were divided into training (80%) and test (20%) sets for the binary classification task of distinguishing between tumor and normal tissue.

For each pair of centers (e.g., centers 0 and 1), we trained HistAuGAN^31^ using the training sets from both centers. A binary classifier was then trained on the training set of one center with HistAuGAN for data augmentation and tested on the test set of the other center. Similarly, we applied Stain SAN on each pair of centers to adapt the training set of one center and the test set of the other center. A binary classifier was then trained on the stain-adapted training set of one center and tested on the adapted test set of the other center. These training and testing processes were repeated for each center pair.

We used ResNet18 as the binary classifier for both stain adaptation methods. The hyperparameters for training the binary classifiers were consistent across both algorithms. The training setup followed this GitHub repository: https://github.com/liucong3/CAMELYON17. Default settings from the following HistAuGAN GitHub repository were used for the hyperparameters when training HistAuGAN: https://github.com/sophiajw/HistAuGAN.

Recall that Stain SAN does not require access to the target domain’s training set for data augmentation or normalization, allowing us to train the binary classifier on all data from one center and test on all data from another center. However, HistAuGAN requires a certain amount of target domain data for training the augmentation tool. Thus, the comparison is inherently biased against Stain SAN as it uses less information in the above setting. Nevertheless, the experimental settings were configured to facilitate a direct comparison between the two algorithms.

We recorded the AUC, accuracy, F1, specificity, and sensitivity of the binary classifiers on the test sets for comparison. The detailed results are presented in Table 4. It shows that Stain SAN can improve the performance of the cross-center binary classification task by a degree that is in line with that of HistAuGAN. This proves the strength of Stain SAN as these comparable results are obtained without having to access the target domain during training. Stain SAN can also be computationally efficient given that it does not require a separate training step of neural networks for applying stain adaptation.

**Table 4:** Binary classification metrics on cross-center analysis using the CAMELYON17 data. Stain SAN achieves performance closely comparable with HistAuGAN despite not using the target domain data during training.

Train	Test	Method	AUC	Accuracy	F1	Specificity	Sensitivity
Center 0	Center 1	HistAuGAN	0.988	0.944	0.943	0.956	0.932
		Stain SAN	0.987	0.937	0.939	0.903	0.971
Center 1	Center 0	HistAuGAN	0.994	0.979	0.979	0.985	0.974
		Stain SAN	0.995	0.980	0.980	0.976	0.985
Center 0	Center 2	HistAuGAN	0.996	0.980	0.980	0.988	0.972
		Stain SAN	0.979	0.948	0.947	0.959	0.937
Center 2	Center 0	HistAuGAN	0.990	0.963	0.962	0.976	0.949
		Stain SAN	0.989	0.956	0.954	0.989	0.923
Center 1	Center 2	HistAuGAN	0.964	0.910	0.909	0.920	0.900
		Stain SAN	0.963	0.909	0.912	0.883	0.935
Center 2	Center 1	HistAuGAN	0.943	0.876	0.865	0.956	0.796
		Stain SAN	0.933	0.862	0.859	0.879	0.845

## 4 Conclusions

In this paper, we show that stain adaptation can provide substantial benefits for machine learning approaches in histopathology. It increases the reliability of trained models for multiple tasks by adjusting stain domains. Different approaches including stain normalization, stain augmentation, and stain mix-up have been developed in past studies, and each method has its own strengths and weaknesses. We proposed the novel stain adaptation method, Stain SAN, which aggregates the benefits while avoiding the pitfalls.

Section 3.3 shows that the four stain adaptation methods well improve the performance of the classification model. This shows that effective stain adaptation can be performed without the need for complex deep learning-based methods. We can also observe that Stain SAN outperformed the previous stain adaptation methods for the cross-dataset clinical ER status classification task using H&E images. Note that the method improved the classification results in both directions of the cross-dataset setting. Adapted images were evaluated by an expert pathologist, and the Stain SAN adapted cores were confirmed to well preserve key cellular structures.

In Sec. 3.6, we demonstrate that Stain SAN achieves results comparable to the state-of-the-art GAN-based approach without requiring separate training for stain adaptation or access to the target domain during training. Stain SAN performed comparably to HistAuGAN, proving its effectiveness without needing a target domain access during training while keeping its computational efficiency.

In future work, we aim to extend Stain SAN with other target distributions. Some possible choices are an elliptical Gaussian distribution and a uniform distribution. It can be beneficial to apply Stain SAN on other datasets and tasks, such as IHC-stained images and survival analysis for cancer patients. Another potential future direction is applying Stain SAN on other benchmark datasets. Some possible choices are Breast Cancer Semantic Segmentation,^32^ MItosis DOmain Generalization,^33^ and Prostate cANcer graDe Assessment.^34^ Moreover, we plan to evaluate variants of Stain SAN using diverse base model architectures, including CycleGAN,^35^ H&E tailored RandAugment,^36^ and Hierarchical Image Pyramid Transformer.^37^

## Data Availability

The archived version of the code described in this paper can be freely accessed through this GitHub repository: https://github.com/taebinkim7/stain-san. The data that support the findings of this study are not openly available due to reasons of sensitivity and are available from the corresponding author upon reasonable request.
